# Temperature-Responsive Separation Membrane with High Antifouling Performance for Efficient Separation

**DOI:** 10.3390/polym16030416

**Published:** 2024-02-01

**Authors:** Tong Ji, Yuan Ji, Xiangli Meng, Qi Wang

**Affiliations:** School of Chemistry and Chemical Engineering, Harbin Institute of Technology, Harbin 150001, China; jitong31099@163.com (T.J.); jiyuan0713@163.com (Y.J.); wangqi_hit@sina.com (Q.W.)

**Keywords:** separation membrane, temperature-responsive, PVDF, PVDF-g-NIPAAm, graphene oxide

## Abstract

Temperature-responsive separation membranes can significantly change their permeability and separation properties in response to changes in their surrounding temperature, improving efficiency and reducing membrane costs. This study focuses on the modification of polyvinylidene fluoride (PVDF) membranes with amphiphilic temperature-responsive copolymer and inorganic nanoparticles. We prepared an amphiphilic temperature-responsive copolymer in which the hydrophilic poly(N-isopropyl acrylamide) (PNIPAAm) was side-linked to a hydrophobic polyvinylidene fluoride (PVDF) skeleton. Subsequently, PVDF-g-PNIPAAm polymer and graphene oxide (GO) were blended with PVDF to prepare temperature-responsive separation membranes. The results showed that temperature-responsive polymers with different NIPAAm grafting ratios were successfully prepared by adjusting the material ratio of NIPAAm to PVDF. PVDF-g-PNIPAAm was blended with PVDF with different grafting ratios to obtain separate membranes with different temperature responses. GO and PVDF-g-PNIPAAm formed a relatively stable hydrogen bond network, which improved the internal structure and antifouling performance of the membrane without affecting the temperature response, thus extending the service life of the membrane.

## 1. Introduction

Membranes serve as pivotal separation tools that are extensively employed in the domains of water refinement, food processing, pharmaceutical purification, and molecular sieving [1,2,3]. Nonetheless, the equilibrium between permeability and selectivity, antifouling efficacy, and membrane lifetime remain the principal limiting factors in membrane technology applications [4,5]. These limitations circumscribe the applicability and operational range of membranes [6]. Antifouling is intrinsically linked to the membrane’s lifespan and the costs associated with separation processes [7,8]. Fouling mitigation strategies encompass mechanical cleaning methods (e.g., backflushing and scraping), the application of targeted chemical agents for foulant removal, ultrasonic cleaning techniques, and membrane modification to enhance fouling resistance [9]. Membrane modification is considered the most foundational and efficacious approach to reduce the fouling of the membrane [10]. Currently, membrane modification techniques are categorized into matrix modifications, which include blending and polymer grafting, and surface modifications, which involve coating and grafting at the surface level [5,11]. Mohammadi et al. incorporated graphene oxide into a three-layer electrostatic spinning composite nanofiltration membrane to improve its hydrophilicity and salt resistance [12]. Yue et al. created an improved GO-TiO_2_/PVDF composite ultrafiltration membrane that enhances water flow and fouling resistance, leading to significantly more efficient and less energy-intensive treatment of slightly polluted groundwater [13]. Kang et al. developed a highly efficient TiO_2_ nanofiber ultrafiltration membrane on a porous titanium base with superhydrophobicity, achieving a high water flow rate and excellent antifouling properties, effectively filtering out small nanoparticles while maintaining high permeability and rejection rates [14]. 

As the application spectrum of membrane separation broadens and operational conditions grow more demanding, the performance expectations for membranes escalate correspondingly. Advances in this field have introduced innovative smart membrane materials, offering fresh perspectives to researchers [15,16]. In recent years, membranes capable of intelligent responses to various stimuli, including temperature [17], pH [18,19], pressure [20], light [21], electricity [22], and magnetic fields [23], have been developed [24]. Such innovations not only diminish membrane operational costs but also broaden their applications [25]. Shen et al. enhanced a hydrophobic PVDF membrane’s filtration efficacy by rendering it both hydrophilic and pH-sensitive through surface modification [26]. Concurrently, Li et al. fabricated a thermally responsive membrane by grafting PNIPAM onto the PVDF membrane’s surface and pores, enabling water flux modulation through adjustments in PNIPAM grafting density [27]. To date, the manufacture of intelligent response membranes usually involves the modification of the membrane surface by the response molecules, which does not improve the pore structure, hydrophilicity, and antifouling properties. The other method is in response to molecular modification of the membrane body, which may damage the mechanical properties of the membrane and reduce its service life [28]. These factors have hindered the widespread practical adoption of intelligent response separation membranes [29,30]. 

In the present investigation, an amphiphilic thermally responsive polymer was blended with graphene oxide to modify polyvinylidene fluoride (PVDF), culminating in the formation of a temperature-responsive separation membrane through immersion precipitation phase inversion. The thermally responsive copolymer PVDF-g-PNIPAAm was synthesized by conjugating poly(N-isopropyl acrylamide) (PNIPAAm) onto the PVDF backbone utilizing an alkali method. Subsequently, PVDF-g-PNIPAAm and graphene oxide(GO) were intermixed with PVDF, and the performance of the resulting membrane was fine-tuned by modulating the grafting density of PVDF-g-PNIPAAm and the concentration of graphene oxide. Through the strategic regulation of the temperature-responsive organic polymer/inorganic particle and the hydrogen bonding between hydrophilic moieties, the membrane’s overall performance was enhanced while endowing it with temperature-responsive characteristics. 

## 2. Materials and Methods

### 2.1. Materials

PVDF was purchased from Shanghai Huayi Sanaifu New Material Co., Ltd. (Shanghai, China). NIPAAm was obtained from Aladdin. Graphene oxide was purchased from Suzhou Hengqiu Technology Co., Ltd. (Suzhou, China). *N*,*N*′-dimethylformamide (AR, DMF) and potassium hydroxide (KOH) were purchased from Tianjin Fengchuan Material Co., Ltd. (Tianjin, China). Azodiisobutyronitrile (AIBN) was purchased from Beijing Bailingwei Technology Co., Ltd. (Beijing, China). Polyvinylpyrrolidone (PVP, K30) and *N*,*N*′-dimethylacetamide (AR, DMAC) were provided by Tianjin Damao Chemical Reagent Factory (Tianjin, China); BSA was purchased from Beijing Biological Technology Co., Ltd. (Beijing, China). Methanol (99.7%) and ethanol (99.7%) were provided by Tianjin Fuyu Fine Chemical Co., Ltd. (Tianjin, China). These chemicals were used as received. All other reagents used in this work were of commercial analytical grade and used without further purification.

### 2.2. Synthesis of PVDF-g-PNIPAAm Polymer by Alkali Treatment

The PVDF powder was added to a 20 wt% KOH alcohol solution (1:1 by the mass ratio of ethanol to water). The mass ratio of PVDF to KOH alcohol solution was 1:10. Subsequently, the PVDF powder was completely wetted by stirring, and then the mixture was stirred in a water bath at 60 °C for 30 min. After the solution was cooled, it was suction-filtered and washed 4 times with distilled water to remove KOH and ethanol from the surface. The product was dried in an oven at 60 °C for 24 h.

The alkali-treated PVDF powder (5.000 g) was added to 60 mL of DMF solvent, and the mixture was heated and stirred in a water bath at 60 °C to completely dissolve. NIPAAm monomer (2.500 g, 5.000 g, and 10.000 g) and initiator AIBN (0.084 g) were added and vacuumed three times in a nitrogen atmosphere repeatedly. Then, the mixture was stirred for 10 h in a water bath at 60 °C in a nitrogen atmosphere. After cooling to room temperature, a large amount of ethanol was added to the mixture. The reaction product was washed repeatedly with distilled water more than 4 times to remove unreacted monomers and dry. 

Three different PVDF-g-PNIPAAm polymers were prepared by controlling the mass ratio of PVDF to NIPAAm at the same reaction temperature, time, and initiator mass ratio. The mass ratios of PVDF to NIPAAm were 2:1, 1:1, and 1:2, respectively, and the corresponding products were named m21, m11, and m12.

### 2.3. Preparation of Temperature-Responsive Separation Membrane

#### 2.3.1. Preparation of PVDF/PVDF-g-PNIPAAm Blending Membrane

Pure PVDF, PVDF/PVDF-g-PNIPAAm membranes were prepared by using the immersion precipitation phase transformation method. DMAc was used as a solvent and PVP was used as a pore-making agent. The content of PVDF was 19.8 wt%, PVDF-g-PNIPAAm was 0.2 wt%, and the content of PVP was 3.0 wt%. The weighed PVDF, PVDF-g-PNIPAAm, and PVP were added to DMAc, and the casting solution was prepared by stirring for 8 h at 60 °C. It was left to defoam for 24 h at room temperature. The cast membrane solution was poured onto a glass plate and scraped with a casting knife to make the membrane. Then, the glass plate with the casting solution was immersed in deionized water (30 °C). The casting solution cures to form a membrane of appropriate size and thickness. Finally, the residual solvent was removed by thorough rinsing with deionized water. The membrane without PVDF-g-PNIPAAm was labeled as M0. The blended membranes containing PVDF-g-PNIPAAm numbered m21, m11, and m12 were labeled as M21, M11, and M12, respectively.

#### 2.3.2. Preparation of PVDF/PVDF-g-PNIPAAm/GO Separation Membrane

The immobilized PVDF content was 19.8 wt%, the PVDF-g-PNIPAAm content was 0.2 wt%, and the PVP content was 3.0 wt%. DMAc was used as the solvent and the solidification bath temperature was 30 °C. The scheme design is shown in Table 1. GO was added in DMAc at ultrasonic for at least 5 h, which facilitated the dispersion of GO. PVDF, PVDF-g-PNIPAAm, and PVP were added to the dispersion and stirred in a water bath at 60 °C for 8 h. The mixture was left to defoam for 24 h at room temperature. The casting solution was poured onto a glass plate and scraped with a casting knife to make the membrane. Then, the glass plate with the casting solution was immersed in deionized water (30 °C). The casting solution cures to form a membrane of appropriate size and thickness. Finally, the residual solvent was removed by thorough rinsing with deionized water.

**Scheme 1:**
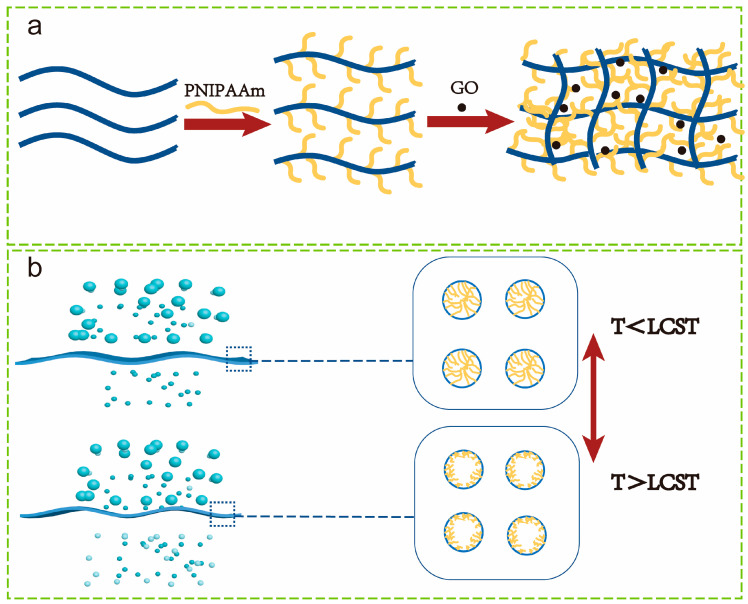
(**a**) Schematic diagram of PVDF/PVDF-g-PNIPAAm/GO membrane formation; (**b**) schematic illustration of temperature response behavior.

The formation process and temperature response behavior of the PVDF/PVDF-G-PNIPAAm/GO membranes are shown in Scheme 1.

### 2.4. PVDF-g-PNIPAAm Characterization

The main functional group of the polymer was analyzed by using a nuclear magnetic resonance spectrometer (AVANCE III, Bruker, Ettlingen, Germany). The grafting ratio of thermo-responsive polymers is calculated from the peak area of the proton characteristic peak. The grafting ratio is calculated as Equation (1).

(1) $$X=\frac{\frac{1}{6}A}{\frac{1}{2}\left(A_{hh}+A_{ht}\right)+\frac{1}{6}A}$$

where *X* is the grafting ratio of PNIPAAm (%), *A* is the peak area of CH(CH_3_)_2_ peak, *A_hh_* is the peak area of hh (-CF_2_-CH_2_-CH_2_-CF_2_-) peak, and *A_ht_* is the peak area of ht (-CF_2_-CH_2_-CF_2_-CH_2_-) peak.

The thermogravimetric method (SDT Q600, TA, New Castle, DE, USA) was used to analyze the thermal properties of the polymers. The sample was heated from room temperature to 800 °C at a rate of 10 °C·min^−1^ in a dry nitrogen atmosphere. 

### 2.5. Membrane Characterization

The mechanical properties of the membranes were measured by using an electronic universal testing machine (Instron 5569, Instron, High Wycombe, UK). The samples were measured at room temperature and a speed of 5 mm·min^−1^. Each group of samples was measured three times and averaged. The mechanical properties of the membrane were analyzed by comparing the Young’s modulus of the membrane. 

The morphologies of the membranes were observed by using a scanning electron microscope (SEM, Instron 5569, Instron, USA). To obtain the cross-section of the membrane, the membrane sample was fractured by immersing it in liquid nitrogen, then it was fixed on a sample stage and was sputtered with gold, and placed into the device for observation. 

The surface hydrophilic property of a membrane was measured by a contact angle goniometer (POWER 2000, Powereach, Shanghai, China) using the sessile drop method. At room temperature, a water droplet was dropped onto the top surface, and the contact angle was recorded every 10 s. The reported data of contact angles were obtained by calculating the average of five repeated measurements. 

### 2.6. Water Permeation Experiments

The pure water flux (*J*_w_) of the ultrafiltration membrane refers to the volume of pure water permeating the membrane in a certain period over a unit membrane area under a certain filtration pressure. 

In this experiment, the water flux of the membrane was measured with a cup ultrafilter (MSC-300, Mosu, Shanghai, China). The ultrafiltration membrane was cut to a size suitable for the filter cup. Before the test, the pressure was statically pressed at 0.1 MPa for 20 min. After the test solution was stabilized, the water flux of the membrane was measured under a pressure of 0.1 MPa, and the volume of water passing through a certain period was recorded to obtain the pure water flux. Water flux (*J*_w_) was calculated by Equation (2):

(2) $$J_{w}=\frac{V}{S\cdot t}$$

where *J*_w_ is the permeation flux of the membrane for pure water (L·h^−1^·m^−2^), *V* is the permeate liquid volume measured (L), *S* is the effective membrane area (m^2^), and *t* is the permeation time (h).

### 2.7. Rejection Property of the Membrane

The rejection rate *R*(%) of the ultrafiltration membrane is indicative of the rejection effect of the ultrafiltration membrane on the bovine serum albumin solution under a certain pressure.

The permeate from the original solution after passing through the ultrafiltration membrane was collected at room temperature and a test pressure of 0.1 MPa. Finally, the absorbance of the original solution of BSA and the permeate at 280 nm was measured with an ultraviolet spectrophotometer. The BSA protein rejection rate was calculated by using Equation (3):

(3) $$R=\left(1-\frac{C_{p}}{C_{f}}\right)\times 100$$

where *R* is the membrane rejection (%), *C*_f_ is the concentration of the original solution (mg·mL^−1^), and *C*_p_ is the permeate concentration (mg·mL^−1^).

### 2.8. Antifouling Properties of the Membrane

The antipollution performance of the ultrafiltration membrane is mainly measured by using the water flux recovery rate *FRR* (%). The ultrafiltration membrane was tested according to the pure water flux test method described above and recorded as *J*_w1_; then, the same membrane was treated with ultrafiltration separation of bovine serum albumin solution for 30 min, and the ultrafiltration membrane was then completely cleaned with distilled water. The membrane was again measured for pure water flux and recorded as *J*_w2_. The ambient temperature was controlled at 22 °C during the experiment. The antipollution performance of the ultrafiltration membrane was investigated by calculating the flux recovery rate from the ratio of the two. The flux recovery rate (FRR) was calculated by using Equation (4):

(4) $$FRR=\frac{J_{W1}}{J_{W2}}\times 100\%$$

where *J*_w1_ is the pure water flux (L·h^−1^·m^−2^) before the use of the ultrafiltration membrane, and *J*_w2_ is the pure water flux (L·h^−1^·m^−2^) after the use of the ultrafiltration membrane.

### 2.9. Porosity Test of the Membrane

The porosity of the membrane was obtained by using the weighing method. The membrane was cut into squares of equal size. The mass of the membrane was weighed separately when dry and after immersion in pure water. The porosity was calculated as shown in Equation (5):

(5) $$P_{r}=\frac{\left(W_{W}-W_{d}\right)}{S\cdot d\cdot \rho }\times 100\%$$

where *W*_w_ and *W*_d_ are the weight of the wet and dry membrane (g), *S* is the area of the measurement membrane (cm^2^), *d* is the average thickness of the membrane (mm), and *ρ* is the density of distilled water at room temperature (g·cm^−3^).

## 3. Results and Discussion

### 3.1. Characterization of the Synthesized PVDF-g-PNIPAAm Polymers

**Figure 1:**
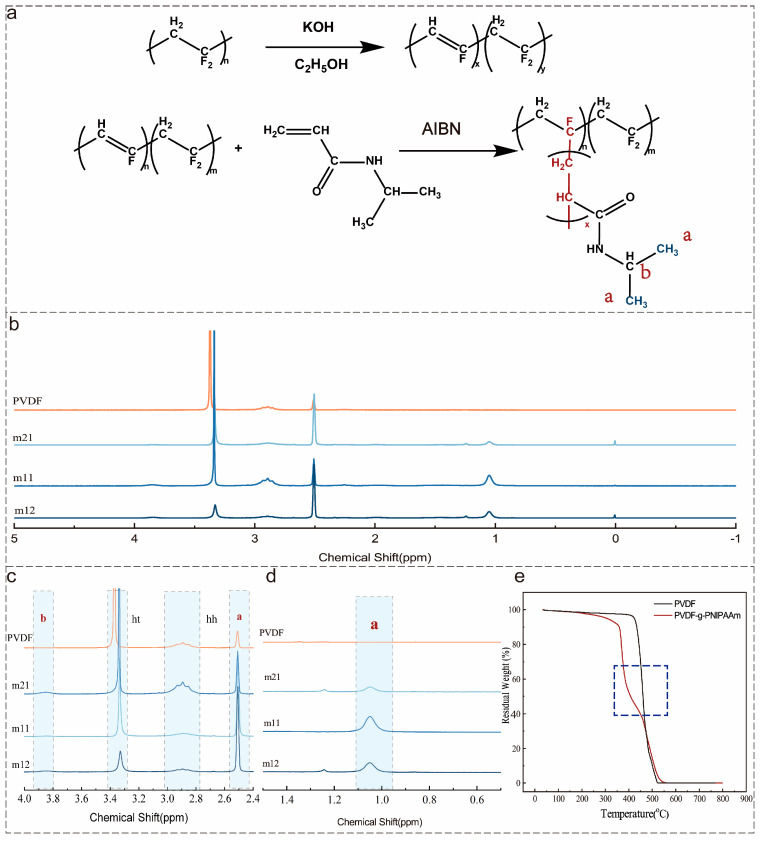
Reaction mechanism, ^1^H NMR spectra, and TG curves of copolymer synthesis: (**a**) reaction mechanism; (**b**) the full ^1^H NMR spectrum of the copolymer; (**c**,**d**) the local spectrum of copolymer ^1^H NMR; (**e**) TG curves of the copolymer.

In this study, NIPAAm monomers were branched onto the hydrophobic PVDF backbone by alkali treatment to prepare the amphiphilic temperature-responsive polymer, the mechanism of which is shown in Figure 1a. The reaction was carried out in two steps. The alkaline alcohol solution removes -F and -H from PVDF to form unsaturated bonds. Next, the initiator AIBN opens C=C in PVDF, and NIPAAm is grafted onto the PVDF skeleton to form a side chain [31]. Three temperature-responsive polymers (m21, m11, and m12) with different NIPAAm grafting ratios were synthesized by adjusting the ratios of PVDF and NIPAAm mass. The ^1^H NMR spectra of PVDF-g-PNIPAAm showed new peaks at δ 1.1 ppm and 3.9 ppm, which were attributed to isopropyl and hypo methyl, respectively, as shown in Figure 1b–1d. In addition, the PNIPAAm grafting ratio of PVDF-g-PNIPAAm can be determined based on the ratio of (-CF_2_-CH_2_-CF_2_-CH_2_-, ht) and (-CF_2_-CH_2_-CH_2_-CF_2_-, hh) peak areas in the PVDF molecular chain with the isopropyl ^1^H NMR peak areas of PNIPAAm (Equation (3)). The grafting ratios of PVDF-g-PNIPAAm were calculated to be 4.9%, 8.3%, and 16.56% (Table 2). 

In addition, the thermal stability of the polymers before and after the reaction was tested, and the heat loss curves are shown in Figure 1e. The thermal weight loss curve of increased a thermal weight loss plateau (360 °C) compared to the pure PVDF thermal weight loss curve, which was attributed to the disconnection of the side chain PNIPAAm. As can be seen, both ^1^H NMR and TG confirmed the successful preparation of PVDF-g-PNIPAAm polymer.

### 3.2. Characterization of PVDF/PVDF-g-PNIPAAm Membrane

**Figure 2:**
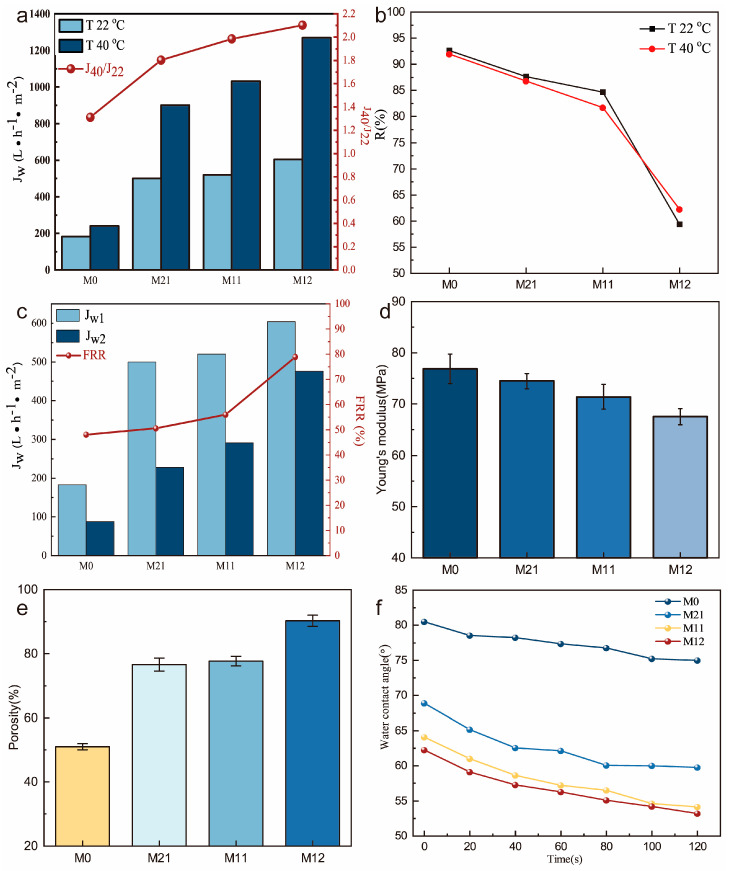
Comprehensive performance of PVDF/PVDF-g-PNIPAAm membranes: (**a**) water flux; (**b**) rejection rate; (**c**) antifouling performance FRR; (**d**) mechanical properties; (**e**) porosity; (**f**) water contact angle.

PVDF-g-PNIPAAm was blended with PVDF to prepare a temperature-responsive membrane. PVP was used as a pore-making agent. Water flux and rejection rates are indicators of membrane separation capacity. In this study, the pure water flux and protein rejection of the PVDF/PVDF-g-PNIPAAm membrane were evaluated at different temperatures. The pure water fluxes of M21, M11, and M12 ranged from 500 to 901 L·m^−2^·h^−1^, 520 to 1032 L·m^−2^·h^−1^, and 604 to 1270 L·m^−2^·h^−1^ when the feed temperature was increased from 22 °C to 40 °C, respectively. In comparison, the pure water flux of the pure PVDF membrane (M0) ranged from 183 to 240 L·m^−2^·h^−1^ when the feed temperature was increased from 22 to 40 °C (Figure 2a). The results show that the pure water flux of the blended membrane increases with the increase in temperature. At the same temperature, the water flux of the membrane increases with the increase in the grafting rate of the PVDF-g-PNIPAAm.

We evaluated the temperature-responsive property of the membrane by the ratio of the water flux at 40 °C and 22 °C (J_40_/J_22_). The temperature response effect increases (J_40_/J_22_) from 1.802 to 2.103 with the increase in the polymer PNIPAAm grafting ratio (4.9% to 16.56%). When the permeate temperature is lower than LCST, the PNIPAAm chains stretch, causing the membrane pores to shrink and even close; and when the permeate temperature is higher than LCST, the PNIPAAm chains contract, causing the membrane pores to be enlarged and the water flux to increase (Scheme 1). When the PNIPAAm grafting ratio increases, the water flux of the blended membrane becomes more dependent on temperature, and thus, the membrane becomes more thermo-responsive. 

The rejection rate is an important indicator of membrane selectivity. Bovine serum protein was used as the standard rejection. As shown in Figure 2b, the rejection of the blended membrane decreased when the PNIPAAm grafting ratio increased at the same temperature. This is because the PNIPAAm chains contain hydrophilic groups. The hydrophilic groups promote the exchange between solvent and non-solvent during the membrane curing process, leaving more holes in the interior of the membrane. When the temperature was increased from 22 °C to 40 °C, the rejection rates of membrane M21 and membrane M11 decreased with increasing temperature. This is due to the contraction of the branch chain of PNIPAAm, the increase in membrane pore size, and the decrease in the rejection rate due to the increase in temperature. 

The M12 membrane has the opposite temperature response behavior to the M21 and M11 membrane. This is due to the high PNIPAAm grafting rate of the M12 membrane. Too many hydrophilic groups cause the pore size to be too large during the membrane curing process. When the temperature is lower than LCST, although the hydrophilic segment of PNIPAAm is in an extended state, the rejection rate is still low because the large pore size makes BSA macromolecules easily pass through the membrane pore. At the same time, the high grafting rate of PNIPAAm leads to the winding of segments on the membrane surface and the membrane pore surface. When the temperature increases, the crimp of the polymer chain does not effectively increase the pore size of the membrane, and the entanglement of the side chain even blocks the pore size of the membrane. The excessive entanglement of PNIPAAm side chains reduces the temperature sensitivity of the blended membranes.

Antifouling performance is a very important property of membranes that determines their service life. The membrane matrix material PVDF has strong hydrophobicity, which can allow it to easily adsorb retained materials and lead to contamination on the membrane surface and pore wall, and excessive contaminants lead to membrane pore blockage and membrane separation failure. As shown in Figure 2c, the antifouling ability of the separation membrane was also improved with the increase in the grafting rate of the temperature-responsive polymer. The increase in the PNIPAAm side chains promotes the antifouling performance of the membrane. This is due to the hydrophilic groups in PNIPAAm inducing water molecules in the filtrate to form an aqueous layer on the membrane surface, which reduces the deposition of separates on the membrane surface. Meanwhile, the increase in membrane hydrophilicity reduces the adsorption of separates by the membrane [32,33].

To further analyze the structure of the temperature-responsive membrane, we characterized the porosity of the membranes. Figure 2e shows the porosity of the blended membranes with different NIPAAm grafting ratios. The PVDF membranes containing only the pore-making agent PVP had a lower porosity of 50.99%. The addition of the polymer significantly increased the porosity of the membrane. This may be attributed to the hydrophilic groups in the amphiphilic polymer inducing water molecules to enter the casting solution faster during the membrane curing process. This accelerated the exchange of soluble and non-soluble components to create larger pores.

In addition, the surface hydrophilicity of the membrane was characterized by water droplet trapping (Figure 2f). The 20 s static water contact angle of the PVDF membrane without temperature-responsive polymer addition was 78.56°. The water contact angles of M21, M11, and M21 would be 65.18°, 61.04°, and 59.13°, respectively. In addition, the water contact angle of the pure PVDF membrane decreased from 80.51° to 75.01° at 120 s. The water contact angle of the co-blended membrane decreased more obviously. After 120 s, the water contact angle of the M21 membrane decreased to 59.78°, the M11 membrane decreased to 54.14°, and the M12 membrane decreased to 53.17°. The results indicated that the hydrophilicity of the membranes increased with the increase in the PNIPAAm grafting ratio. This is consistent with the above characterization results [32].

However, we found that the addition of PVDF-g-PNIPAAm caused a decrease in the mechanical properties of the membranes. As shown in Figure 2d, the Young’s modulus of the blend membrane decreased with the increase in the PNIPAAm grafting ratio. It demonstrates that the addition of PVDF-g-PNIPAAm increases the porosity of the membrane and the internal structure of the membrane becomes loose. It causes the membrane to reduce its ability to resist external forces. It also shows that the rejection rate of the blend membrane decreases sharply when the grafting ratio of PNIPAAm is much too high.

This suggests that the temperature-responsive polymer has some improvement in the separation performance of the membrane while imparting a temperature-responsive function to the membrane. When the grafting rate of PVDF-g-PNIPAAm was 8.3%, the temperature response property of the separation membrane was the best. However, the enhancement of the overall performance of the membrane has limitations.

### 3.3. Characterization of PVDF/PVDF-g-PNIPAAm/GO Membrane

**Figure 3:**
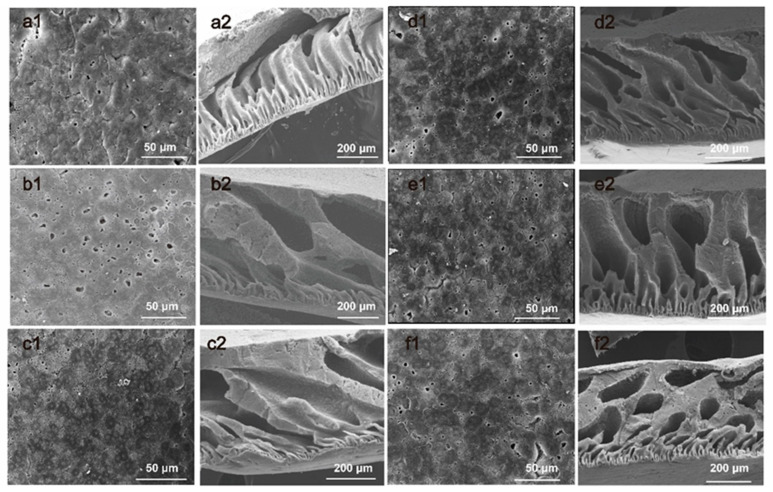
SEM photographs of the membranes: (**a1**–**f1**) the upper surfaces of pure PVDF membranes, PVDF/PVDF-g-PNIPAAm membranes, 0.25 wt%, 0.50 wt%, 0.75 wt%, 1.00 wt% GO content PVDF/PVDF-g-PNIPAAm/GO membranes; (**a2**–**f2**) the cross-section of the pure PVDF membranes, PVDF/PVDF-g-PNIPAAm membranes, 0.25 wt%, 0.50 wt%, 0.75 wt%, 1.00 wt% GO content PVDF/PVDF-g-PNIPAAm/GO membranes.

In order to improve the overall performance of the membrane without destroying the temperature-responsive property, we considered introducing inorganic nanoparticles into the membrane. The PVDF-g-PNIPAAm and GO were added together to PVDF to prepare the PVDF/PVDF-g-PNIPAAm/GO separation membrane. Figure 3 shows SEM photographs of different separation membranes, which include the upper surface and cross-section of the membrane. It included PVDF/PVDF-G- PNIPAAm/GO membranes containing 0.25 wt%, 0.50 wt%, 0.75 wt%, and 1.00 wt% GO. It is easy to establish that the incorporation of hydrophilic GO significantly changed the membrane pore structure. The surface of pure PVDF ultrafiltration membrane is smooth, and the number of pores is small and uniformly distributed. The addition of the PVDF-g-PNIPAAm increased the pore size of the membrane. However, it was still unevenly distributed, and the internal pores were mostly spongy. With the addition of graphene oxide, the surface roughness of the membrane increased, the pore size distribution narrowed, the pore distribution became more uniform, and more finger-like pores formed in the membrane.

**Figure 4:**
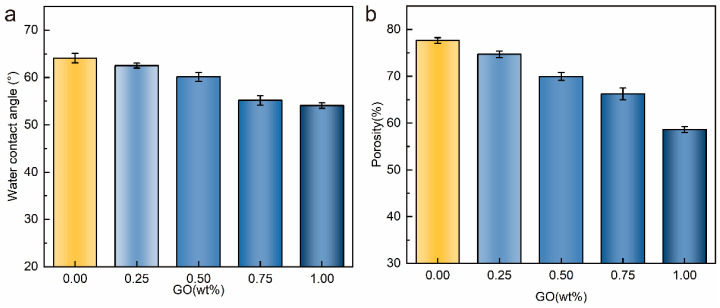
Porosity and hydrophilicity of PVDF/PVDF-g-PNIPAAm/GO membrane: (**a**) porosity; (**b**) hydrophilicity.

Figure 4 shows the test results of hydrophilicity and porosity of the membranes. Figure 4a shows the hydrophilic characterization of the membrane after adding GO. We found that the addition of GO significantly improved the hydrophilicity of the membrane surface. This is because graphene oxide is rich in hydrophilic groups. When the casting solution came into contact with water molecules, the GO attracted the water molecules, and eventually, the GO accumulated on the surface of the membrane. The amount and roughness of hydrophilic groups on the surface of the membrane were increased by graphene oxide, which improved the hydrophilicity of the membrane. However, in the characterization of membrane porosity, we found that the addition of GO containing rich hydrophilic groups caused the porosity of the membrane to decrease instead of increase (Figure 4b). Combined with the SEM photograph, after GO was added, the inside of the membrane changed from large pores to more small pores (Figure 3). This is due to the fact that the hydrophilic groups on the GO surface and the PNIPAAm chains are connected by hydrogen bonds into a tighter network structure. The dense hydrogen bond network impedes the exchange rate between the water molecules entering the membrane and the solvent. It promotes the formation of uniform small aperture finger pores.

**Figure 5:**
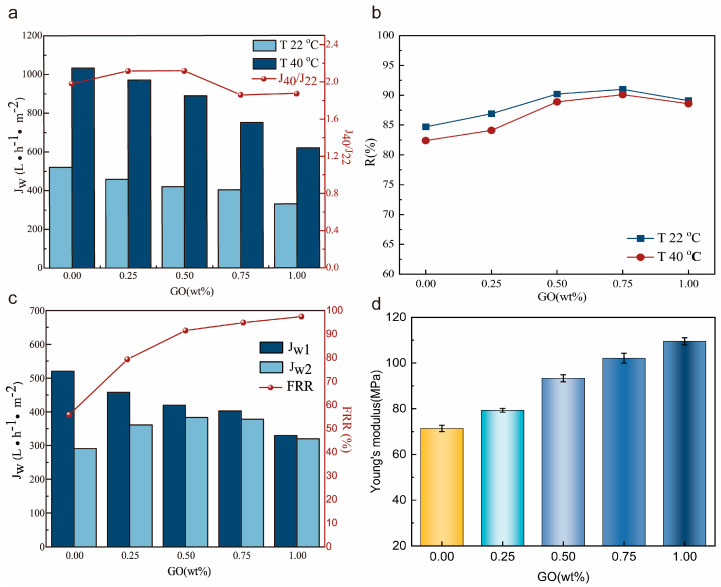
Comprehensive performance of PVDF/PVDF-g-PNIPAAm/GO membranes: (**a**) water flux; (**b**) rejection rate; (**c**) antifouling performance FRR; (**d**) mechanical properties.

We also used the ratio of water flux at 40 °C and 22 °C to evaluate the temperature-sensitive performance of the membranes, and the addition of graphene oxide has little effect on the temperature response of the membranes (Figure 5a). At the same time, the membrane rejection rate also increases with the addition of GO (Figure 5b). Combined with the membrane microstructure, we believe that the increase in the retention rate is due to the decrease in the number of large pore sizes and the decrease in the overall porosity, which restricts the passage of macromolecular proteins. In the antifouling test, it can be seen that the addition of GO significantly improves the antifouling performance of the membrane, as shown in Figure 5c. This is due to the increased hydrophilicity of the membrane surface, which reduces the deposition of contaminants on the membrane surface. In addition, the mechanical properties of the membranes were measured by using tensile properties. The addition of GO increased the Young’s modulus of the membrane, which indicates that the structure of the membrane is tighter and can withstand more damage from external forces (Figure 5d). Overall, the addition of GO successfully achieved the antifouling and mechanical properties of the membrane. This is conducive to improving the service life of the membrane.

## 4. Conclusions

Temperature-responsive separation membranes have made significant contributions to the field of water treatment. However, it is very challenging to provide a membrane with temperature-sensitive properties while improving its separation efficiency and antifouling performance. In this study, we successfully prepared an amphiphilic temperature-responsive polymer, PVDF-g-PNIPAAm, with varying grafting rates. Subsequently, we used these functional polymers to create temperature-responsive separation membranes. By adjusting the PNIPAAm grafting rate of the polymer, we further enhanced the temperature response of the membrane. Moreover, we successfully improved the pore structure both on the membrane surface and inside the membrane through hydrogen bond cross-linking between graphene oxide and PNIPAAm. By doing so, we were able to improve the separation efficiency and lifespan of the membrane without affecting its temperature response. The structural combination of the polymer chain and inorganic particles, as well as the interaction between hydrophilic groups, presented in this study provide a novel approach for the preparation of intelligent response separation materials with excellent comprehensive properties. In the future, it may be possible to incorporate various stimulus-responsive molecules and utilize the multidimensional structure inorganic particles to create a stable membrane structure. This will not only expand the application range of membrane materials but also further improve the overall properties of the membrane.

## Figures and Tables

**Table 1:** Preparation parameters of PVDF/PVDF-g-PNIPAAm/GO blending membranes.

Membranes	PVDF (wt%)	PVDF-g-PNIPAAm (wt%)	GO (wt%)
1	19.8	0.20	0.00
2	19.8	0.20	0.25
3	19.8	0.20	0.50
4	19.8	0.20	0.75
5	19.8	0.20	1.00

**Table 2:** Grafting rates of PNIPAAm in polymers of different mass ratios.

Number	The Mass Ratio of PVDF to NIPAAm	X_PNIPAAm_ (%)
m_21_	2:1	4.9
m_11_	1:1	8.3
m_12_	1:2	16.56

## Data Availability

Data are contained within the article.
